# Ubiquitin–Proteasome-Dependent Regulation of Bidirectional Communication between Plastids and the Nucleus

**DOI:** 10.3389/fpls.2017.00310

**Published:** 2017-03-15

**Authors:** Yoshihiro Hirosawa, Yasuko Ito-Inaba, Takehito Inaba

**Affiliations:** ^1^Department of Agricultural and Environmental Sciences, Faculty of Agriculture, University of MiyazakiMiyazaki, Japan; ^2^Organization for Promotion of Tenure Track, University of MiyazakiMiyazaki, Japan

**Keywords:** proteasome, ubiquitin, plastid protein import, plastid biogenesis, retrograde signaling

## Abstract

Plastids are DNA-containing organelles and can have unique differentiation states depending on age, tissue, and environment. Plastid biogenesis is optimized by bidirectional communication between plastids and the nucleus. Import of nuclear-encoded proteins into plastids serves as anterograde signals and vice versa, plastids themselves send retrograde signals to the nucleus, thereby controlling *de novo* synthesis of nuclear-encoded plastid proteins. Recently, it has become increasingly evident that the ubiquitin–proteasome system regulates both the import of anterograde plastid proteins and retrograde signaling from plastids to the nucleus. Targets of ubiquitin–proteasome regulation include unimported chloroplast precursor proteins in the cytosol, protein translocation machinery at the chloroplast surface, and transcription factors in the nucleus. This review will focus on the mechanism through which the ubiquitin–proteasome system optimizes plastid biogenesis and plant development through the regulation of nuclear–plastid interactions.

On the other hand, plastids also send feedback signals to regulate the expression of genes encoding plastid proteins in the nucleus. These signals are known as retrograde signals from plastids to the nucleus and are referred to as plastid signals. Plastid signals can be divided into two types: biogenic and operational (Pogson et al., 2008). Among them, biogenic signals are necessary to coordinate gene expression in two genomes, allowing cells to assemble the photosynthetic apparatus and to promote chloroplast development (Pogson et al., 2008; Inaba et al., 2011; Jarvis and Lopez-Juez, 2013). To date, several transcription factors have been shown to mediate biogenic signals from plastids to the nucleus (Koussevitzky et al., 2007; Ruckle et al., 2007; Kakizaki et al., 2009; Waters et al., 2009; Sun et al., 2011; Martin et al., 2016).

A number of studies have demonstrated the roles of *de novo* synthesis and the targeting of plastid precursor proteins in the regulation of nuclear–plastid interactions. However, it has become increasingly evident that the nuclear–plastid interaction is also regulated by the degradation of multiple components through the ubiquitin–proteasome system (Lee et al., 2013; Ling and Jarvis, 2015). Here, we focus on recent advances in our understanding of how the ubiquitin–proteasome system regulates the nuclear–plastid interaction and plastid biogenesis. Other comprehensive reviews cover broad aspects of plastid protein import and plastid signaling (Li and Chiu, 2010; Inaba et al., 2011; Jarvis and Lopez-Juez, 2013; Pfannschmidt and Munné-Bosch, 2013; Paila et al., 2015; Chan et al., 2016), and space limitations prevent us from providing adequate coverage of all aspects of nuclear–plastid interaction.

## Degradation of Unimported Chloroplast Precursor Proteins by the Ubiquitin–Proteasome Pathway

**FIGURE 1:**
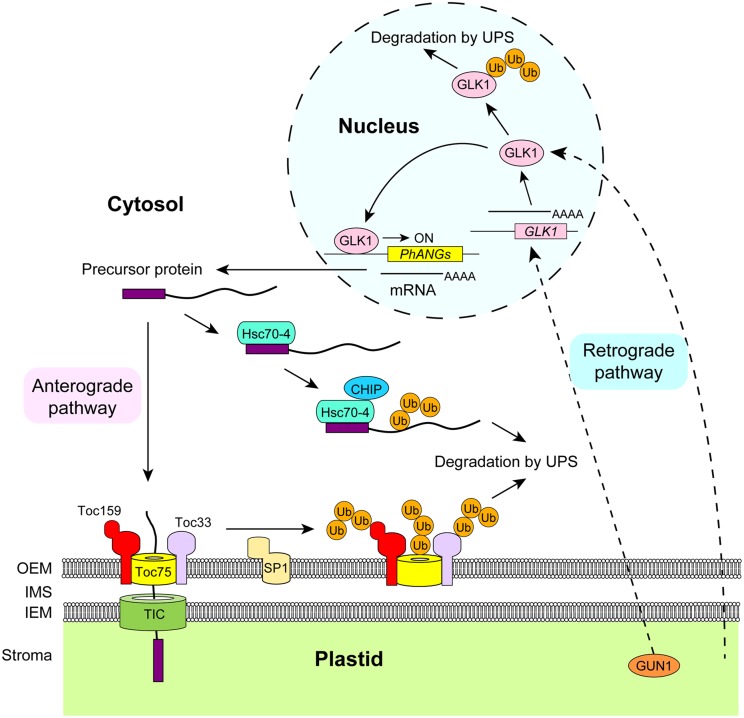
**Control of bidirectional signaling between plastids and the nucleus by the ubiquitin–proteasome system.** Chloroplast development is promoted by the expression of nuclear-encoded *PhANGs* and the import of their products into chloroplasts. When excess precursors are produced, they are recognized by the heat shock cognate 70-4 (Hsc70-4) complex in the cytosol. Subsequently, they are polyubiquitinated by an E3 ubiquitin ligase, carboxy terminus of Hsc70-interacting protein (CHIP), resulting in their degradation by the proteasome. The translocon at the outer envelope membrane of chloroplasts (TOC) complex is also directly targeted by the ubiquitin proteasome system. At least three TOC components, Toc159, Toc75, and Toc33, are polyubiquitinated by a membrane-anchored E3 ubiquitin ligase, suppressor of *ppi1* locus1 (SP1). To further optimize the amount of protein import into chloroplasts, retrograde signals from chloroplasts regulate the level of the GOLDEN2-LIKE 1 (GLK1) transcription factor in the nucleus. Polyubiquitination of GLK1 is induced when chloroplast biogenesis is inhibited. The degradation of GLK1 results in the down-regulation of *PhANGs*, thereby preventing the accumulation of unnecessary precursor proteins in the cytosol. GLK1 is also regulated by retrograde signals at transcriptional level, and this regulation is mediated by GENOMES UNCOUPLED 1 (GUN1). Although this figure proposes a model for photosynthetic tissues, similar regulation by the ubiquitin–proteasome system appears to play key roles in plastid development in other tissues. Note that a number of other pathways between plastids and the nucleus have been identified, and those pathways are not shown in this figure due to space limitations but can be found in other adequate reviews. UPS, ubiquitin–proteasome system; OEM, outer envelope membrane; IEM, inner envelope membrane; IMS, intermembrane space; Ub, ubiquitin; *PhANGs*, photosynthesis-associated nuclear genes.

It is well known that the expression of nuclear-encoded photosynthesis-associated genes are induced upon illumination and that mass transport of proteins encoded by these genes into plastids are indispensable for chloroplast development. Those plastid-targeted proteins are encoded as precursors in the nucleus, but only mature proteins are detectable under normal conditions *in vivo*. To avoid the accumulation of unimported proteins in the cytosol, plants have evolved at least two distinct mechanisms. One is feedback regulation of nuclear gene expression by plastid-derived signals, and the other is degradation of unimported precursor proteins by the ubiquitin–proteasome system (Lee et al., 2013; **Figure 1**).

Cytosolic heat shock cognate 70-4 (Hsc70-4) and carboxy terminus of Hsc70-interacting protein (CHIP) appear to be involved in the degradation of unimported precursor proteins in *Arabidopsis thaliana* (Lee et al., 2009). Hsc70-4 recognizes specific sequence motifs within the transit peptide of ribulose-1,5-bisphosphate carboxylase/oxygenase small subunit protein and light-harvesting chlorophyll *a*/*b*-binding protein. Subsequently, CHIP interacts with Hsc70-4 and serves as an E3-ubiquitin ligase, thereby allowing unimported precursors to be degraded through the ubiquitin–proteasome system. This suggests that a transit peptide may function as both a chloroplast targeting signal and a degradation signal when unimported precursors accumulate in the cytosol. This idea is further substantiated by the findings of a recent proteomic study (Sako et al., 2014), in which certain plastid precursors were shown to interact with the proteasome both *in vivo* and *in vitro*.

The mechanism that discriminates between plastid-targeted precursors and proteasome-targeted precursors remains to be characterized in detail. Intriguingly, the *plastid protein import 2* (*ppi2*) mutant of *A. thaliana*, which lacks the atToc159 protein import receptor of plastids, accumulated *N*-acetylated plastid precursor proteins outside of plastids (Bischof et al., 2011). Although atToc159 plays key roles in the import of photosynthesis-associated proteins into plastids, it also participates in the import of constitutively expressed plastid proteins. As will be discussed later, the *ppi2* mutant has been known to exhibit down-regulation of genes encoding photosynthesis-associated proteins in the nucleus in response to plastid-derived signals, but not the expression of constitutive plastid proteins. In contrast, some constitutively expressed proteins were shown to be *N*-acetylated in the *ppi2* mutant. It has been shown that *N*-acetylation serves as a degradation signal for the ubiquitin–proteasome system in yeast (Hwang et al., 2010). Hence, one can speculate that excess precursors that cannot be controlled at the transcriptional level are subjected to *N*-acetylation and ubiquitin–proteasome-dependent degradation. As such, degradation of excess plastid precursors via the ubiquitin–proteasome system plays a key role in determining the amount of protein import and plastid biogenesis.

## Plastid Protein Import Machinery is a Direct Target of Ubiquitin–Proteasome Pathway

The ubiquitin–proteasome system directly regulates the protein translocation machinery at the plastid surface (Ling and Jarvis, 2015; **Figure 1**). This unexpected link was demonstrated in an attempt to isolate a suppressor mutant of *plastid protein import 1* (*ppi1*). The *ppi1* mutant of *A. thaliana* exhibits a pale green phenotype due to the lack of atToc33 in the TOC complex, but can survive on soil. One suppressor mutant of *ppi1*, designated as *suppressor of ppi1 locus1* (*sp1*), possesses a lesion within the RING-type ubiquitin E3 ligase gene (Ling et al., 2012). TOC components are more abundant (1.5- to 2-fold) in the *sp1* mutant than in the wild-type. The wild-type SP1 protein interacts with components of TOC machinery. Furthermore, atToc159, atToc75, and atToc33 have been shown to be polyubiquitinated by SP1 activity. These findings indicate that the ubiquitin–proteasome system directly regulates the level of TOC components, thereby affecting the amount of protein import into plastids.

This mechanism also seems to play a key role in determining the fate of plastids within the cell (Ling et al., 2012). During the photomorphogenic response, the *sp1* single mutant displayed inefficient de-etiolation with reduced levels of photosynthesis-associated proteins and imbalanced TOC receptor levels. The *sp1* mutant also exhibited delayed senescence, and this was accompanied by the delayed transformation from chloroplasts to gerontoplasts within the cell. In contrast, overexpression of SP1 accelerated both de-etiolation and senescence. Hence, regulation of TOC components by the ubiquitin–proteasome system appears to be indispensable for determining both the quality and the quantity of plastid-targeted proteins, thereby affecting the fate of plastid and plant development.

## Regulation of Plastid-to-Nucleus Retrograde Signaling Via the Ubiquitin–Proteasome Pathway

In addition to the anterograde signaling pathway, a recent study demonstrated that the retrograde signaling pathway from plastids to the nucleus is also subjected to ubiquitin–proteasome-dependent regulation in *A. thaliana* (Tokumaru et al., 2017). The key mechanism involves the regulation of the GOLDEN2-LIKE 1 (GLK1) transcription factor by the ubiquitin–proteasome system (**Figure 1**).

The GLK family of transcription factors was originally isolated in maize (Hall et al., 1998; Rossini et al., 2001). The *GLK* genes positively regulate the expression of photosynthesis-associated genes in numerous plants, thereby strongly promoting chloroplast development (Fitter et al., 2002; Yasumura et al., 2005; Waters et al., 2009). Overexpression of *GLK* has been shown to be sufficient to induce chloroplast development in rice calli (Nakamura et al., 2009) and *Arabidopsis* root cells (Kobayashi et al., 2012; Tokumaru et al., 2017). Two separate studies reported that the expression of *GLK* genes responds to inhibitor treatment thus compromising chloroplast development (Kakizaki et al., 2009; Waters et al., 2009). The findings of those studies concluded that *GLK* gene expression responds to plastid signals, resulting in the regulation of photosynthesis-associated genes in response to plastid signals. Intriguingly, impaired chloroplast development caused by the *ppi2* mutation also suppress *GLK1* expression in the nucleus (Kakizaki et al., 2009). This regulation is mediated by the retrograde signaling pathway, because the GENOMES UNCOUPLED 1 (GUN1) protein, which is localized in plastids, appears to act upstream of *GLK1*. From those studies, it becomes clear that plastids transmit signals to determine the amount of anterograde protein import, thereby avoiding the accumulation of excess levels of precursors within the cytosol.

Besides transcriptional regulation, a recent study showed that plastid signals also directly regulate the level of GLK1 protein (Tokumaru et al., 2017). The *GLK1* gene is fully expressed in *gun1* mutants treated with norflurazon. In contrast, the level of GLK1 protein is much lower than that expected from the *GLK1* mRNA levels in the norflurazon-treated *gun1* mutant. The discrepancy between GLK1 protein and mRNA levels is in part attributable to the degradation of the GLK1 protein by the ubiquitin–proteasome system (Tokumaru et al., 2017). When norflurazon-treated plants were further treated with MG-132, a proteasome inhibitor, the accumulation of GLK1 was partially restored. Because the *gun1* mutant also exhibited the same response, it appears that GUN1 is not required for the proteasome-mediated regulation of GLK1. Likewise, MG-132 treatment partially restored the level of GLK1 protein in the *ppi2* mutant. Hence, this mechanism is also used to optimize the expression of nuclear genes encoding photosynthesis-associated proteins when plastid protein import is compromised.

The level of GLK is also regulated by the ubiquitin–proteasome system in fruit tissues of tomato. The *Solanum lycopersicum* GLK2 protein, SlGLK2, regulates chloroplast development in tomato fruit tissues, and fruits of the *slglk2* mutant exhibit uniformly light green coloration (Powell et al., 2012; Nguyen et al., 2014). SlGLK2 was found to be degraded by the ubiquitin E3 ligase complex containing CULLIN4 (CUL4) and UV-DAMAGED DNA BINDING PROTEIN 1 (DDB1; Tang et al., 2016). Consistent with this observation, a mutation in DDB1 significantly increased the pigment contents and chloroplast/chromoplast size in tomato fruits (Cookson et al., 2003), presumably due to the excess accumulation of SlGLK2. Although the roles of SlGLK2 in retrograde signaling remains to be established, these studies further support the idea that the ubiquitin–proteasome pathway is indispensable for the regulation of GLKs.

## Conclusion and Perspective

Although the *de novo* synthesis and targeting of plastid precursor proteins are indispensable for plastid biogenesis, it becomes clear that ubiquitin–proteasome-dependent protein degradation also plays a key role in the regulation of plastid biogenesis. Meanwhile, a number of questions remains to be solved: Are there any other ubiquitin–proteasome regulated transcription factors involved in the retrograde signaling from plastids to the nucleus? Is ubiquitin–proteasome system indispensable for the retrograde signaling from plastids other than chloroplasts? Does operational control of retrograde signaling also requires ubiquitin–proteasome system? In fact, other studies start addressing these questions. Proteasome-regulated transcription factors, such as ELONGATED HYPOCOTYL 5 (HY5) and PHYTOCHROME INTERACTING FACTORS (PIFs), have been shown to participate in retrograde signaling, as well as in light signaling (Ruckle et al., 2007; Martin et al., 2016). Reactive oxygen species-producing chloroplasts appear to be ubiquitinated and subsequently degraded (Woodson et al., 2015). Further investigation will provide novel insight into the roles of the ubiquitin–proteasome system in regulating plastid biogenesis and plant development.

## Author Contributions

TI wrote the manuscript with assistance of YH and YI-I.

## Conflict of Interest Statement

The authors declare that the research was conducted in the absence of any commercial or financial relationships that could be construed as a potential conflict of interest.
